# Bisphenol A Regulates Sodium Ramp Currents in Mouse Dorsal Root Ganglion Neurons and Increases Nociception

**DOI:** 10.1038/s41598-019-46769-6

**Published:** 2019-07-16

**Authors:** Sergi Soriano, Minerva Gil-Rivera, Laura Marroqui, Paloma Alonso-Magdalena, Esther Fuentes, Jan-Ake Gustafsson, Angel Nadal, Juan Martinez-Pinna

**Affiliations:** 1 Departamento de Fisiología, Genética y Microbiología, Universidad de Alicante, Alicante, Spain; 2 Institute of Research, Development and Innovation in Biotechnology of Elche (IDiBE), Institute of Molecular and Cellular Biology (IBMC) and CIBERDEM, Miguel Hernández University of Elche, Elche, Alicante Spain; 3 Department of Biology and Biochemistry, Center for Nuclear Receptors and Cell Signaling, University of Houston, Houston, Texas USA; 4 Department of Biosciences and Nutrition, Karolinska Institut, Huddinge, Sweden

**Keywords:** Neurophysiology, Ion channels in the nervous system

## Abstract

17β-Estradiol mediates the sensitivity to pain and is involved in sex differences in nociception. The widespread environmental disrupting chemical bisphenol A (BPA) has estrogenic activity, but its implications in pain are mostly unknown. Here we show that treatment of male mice with BPA (50 µg/kg/day) during 8 days, decreases the latency to pain behavior in response to heat, suggesting increased pain sensitivity. We demonstrate that incubation of dissociated dorsal root ganglia (DRG) nociceptors with 1 nM BPA increases the frequency of action potential firing. *SCN9A* encodes the voltage-gated sodium channel Na_v_1.7, which is present in DRG nociceptors and is essential in pain signaling. Na_v_1.7 and other voltage-gated sodium channels in mouse DRG are considered threshold channels because they produce ramp currents, amplifying small depolarizations and enhancing electrical activity. BPA increased Na_v_-mediated ramp currents elicited with slow depolarizations. Experiments using pharmacological tools as well as DRG from ERβ^−/−^ mice indicate that this BPA effect involves ERα and phosphoinositide 3-kinase. The mRNA expression and biophysical properties other than ramp currents of Na_v_ channels, were unchanged by BPA. Our data suggest that BPA at environmentally relevant doses affects the ability to detect noxious stimuli and therefore should be considered when studying the etiology of pain conditions.

## Introduction

Bisphenol A (BPA) is an endocrine-disrupting chemical that acts as a xenoestrogen, among other mechanisms of action^1^. BPA is produced in large quantities in the manufacture of polycarbonate plastics and epoxy resins and it is released from common household materials, including polycarbonate plastics, the lining of food cans and thermal paper, which may explain why 93% of the U.S. population presented measurable amounts of BPA in their urine^2^. BPA exposure has been associated with several hormone-related diseases, including obesity and diabetes, female and male reproductive alterations, hormone-sensitive cancers, thyroid hormone level disruption and important alterations of the nervous system^3^.

One important effect of BPA on the nervous system, which remains poorly understood, is the alteration of pain sensation. Behavioral *in vivo* studies reported that male and female rats perinatally treated with 40 μg/kg/day BPA (an environmentally relevant dose orally administered) presented increased pain sensitivity or hyperalgesia^4^, indicating that nociceptive neurons may be altered. As reported in this previous study, BPA increased the frequency with which animals lick an area of the body injured by a formalin injection. This is probably a supraspinal-mediated response involving opioid circuits of the limbic system; however, a direct action of BPA on nociceptors could also be involved. In female rats, the ability of BPA to increase estradiol plasma levels in female rats could have contributed to the effects. In a multibehavioral *in vivo* model of migraine, treatment of adult ovariectomized rats with 500 μg/kg/day BPA (intraperitoneally administered) exacerbates migraine-like behavior, including locomotor activity, light and sound aversion and grooming (indicating facial allodynia). Importantly, alongside these symptoms of nociceptive hypersensitivity, trigeminal neurons showed an increased mRNA expression of estrogen receptors, extracellular signal–regulated kinases (ERK1/2) and Na_v_1.8 encoding Na^+^ channels^5^. However, 500 μg/kg/day of BPA intraperitoneally administered may not be considered an environmentally relevant dose^6^. As a conclusion, although BPA seems to be involved in altered nociception, no study has yet investigated the BPA effects on nociceptors *in vitro* at doses that are relevant to human exposure.

Numerous studies on both animals and humans have demonstrated that the natural hormone 17-β estradiol (E_2_) regulates pain sensitivity and plays a role in gender differences^7^. A series of studies have unveiled at least five molecular mechanisms involved in this E_2_ effect. Firstly, an increased glutamate N-methyl-D-aspartate (NMDA) receptor expression and phosphorylation in spinal neurons processing visceral nociception^8^. Secondly, regulation of P2X2-mediated peripheral pain by acting on estrogen receptors α (ERα) and GPR30 receptors expressed in primary afferent neurons through the cyclic adenosine monophosphate (cAMP)-protein kinase A (PKA)-ERK1/2 intracellular pathway^9^. Thirdly, potentiation of acid-sensing ion channels in DRG neurons via an ERα and ERK1/2 signaling pathway^10^ Fourthly, upregulation of transient receptor potential vanilloid 1 (TRPV1) channels via E_2_-induced genomic and nongenomic mechanisms^11^ and fifthly, upregulated the expression of voltage-gated sodium channels Na_v_1.7, probably through ERα^12^. Therefore, given that E_2_ affects pain behavior, it is interesting to further characterize the actions of BPA, acting as a xenostrogen, on pain. Indeed previous results of our group have shown that BPA acts as a potent estrogen within the 1–100 nM range in excitable cells, e.g., pancreatic β-cells, via extranuclear estrogen receptors ERα and ERβ^13,14^. In pancreatic β-cells, BPA acts through extranuclear ERα and ERβ to modify the shape of action potentials as well as electrical activity which result from altered expression and biophysical properties of voltage-gated calcium-channels^15^. Other changes to ion channel activity have been observed in response to BPA, although normally using high doses within the 10–100 µM range^16–19^.

In the present study, we show that adult male mice treated with 50 μg/kg/day BPA for 8 days presented increased pain sensitivity to heat. To understand how BPA exacerbated pain sensation, we isolated DRG neurons and studied their electrical activity. We found that 1 nM BPA increased the frequency of action potential firing of DRG neurons in response to current injection by increasing the magnitude of Na_v_ ramp currents. Furthermore, the natural hormone E_2_ (1 nM) increased the magnitude of ramp current to a similar extent. Na_v_1.7 sodium channels play a pivotal role in initiating action potentials in response to depolarization of sensory neurons by noxious stimuli^20^, and loss-of-function mutations of the underlying gene, *SCN9A*, provoke an inability to feel pain^21^. In contrast, gain-of-function mutations of *SCN9A* cause severe pain disorders such as inherited erythromelalgia and paroxysmal extreme pain disorder^22^. The effect of BPA on Na_v_ ramp currents reported here was mediated by the activation of the estrogen receptor ERα in a pathway involving phosphoinositide 3-kinase (PI3K).

## Results

### BPA treatment increases thermal nociception

**Figure 1:**
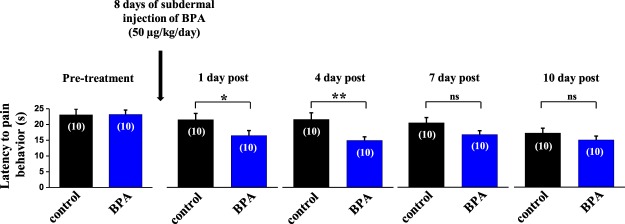
BPA treatment enhances the thermal nociception. Bar graphs showing the average latency of pain reflexes using the hot plate at a fixed temperature of 50 °C in control animals (black bars, n = 10) and animals treated with subdermal injection of BPA (50 µg/kg/day, blue bars, n = 10) for 8 days. Control animals were injected with the same volume of vehicle as the BPA group each day (100 μL of corn oil). Data are represented as the mean ± s.e.m (N = 20 animals). One-way ANOVA for repeated measures: *p < 0.05; **p < 0.01; ns, not significant. (1 day post: p = 0.046; 4 day post: p = 0.007; 7 day post: p = 0.073; 10 day post: p = 0.27).

To analyze if BPA had an action on pain sensitivity *in vivo*, we used adult mice treated with subcutaneous injection of either 50 µg/kg/day BPA or vehicle for 8 days. We observed that the latency to the onset of the pain response following exposure to a metallic hot plate at 50 °C decreased in BPA-treated animals (Fig. 1). One day post-treatment, the delay was 14.3 ± 1.1 s, n = 10 for BPA exposure, compared to 20.7 ± 2 s, n = 10 for controls (p < 0.05; Fig. 1). This BPA-induced reduction was maintained for 4 days after the end of the treatment (Fig. 1). It should be noted that the vehicle on its own caused a small reduction in the latency of the pain response, as previously reported, thus BPA exerted an action in addition to this effect^23^.

### BPA increases excitability in dorsal root ganglion neurons

**Figure 2:**
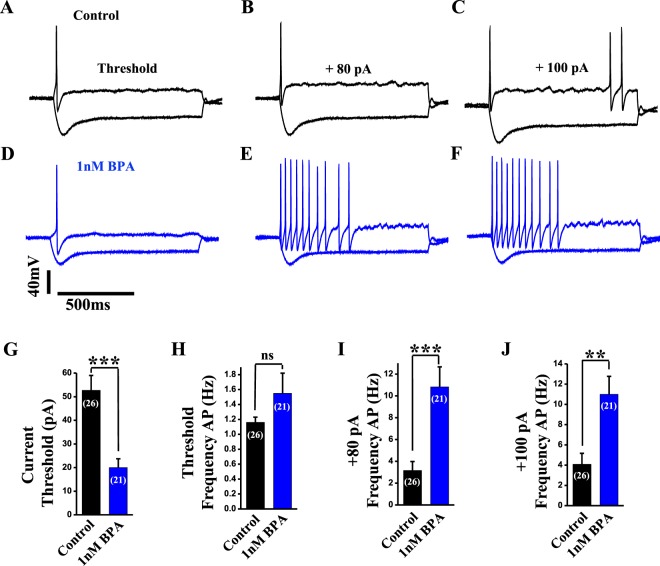
BPA treatment increases excitability in DRG neurons. Representative voltage traces from small dorsal root ganglion neurons in current-clamp experiments in control conditions (**A–C**; black traces) and in the presence of 1 nM BPA for 24–48 h (**D–F**; blue traces) in response to different levels of current injection (threshold, +80 and +100 pA). (**G**) Bar graph showing the average current threshold (pA) for action potential firing in control (n = 26 cells, black bar) and in 1 nM BPA-treated neurons (n = 21 cells, blue bar). (**H**) Bar graph showing the average frequency of action potential firing (Hz) at current threshold injection in control (n = 26 cells, black bar) and in 1 nM BPA-treated neurons (n = 21 cells, blue bar). (**I**) Bar graph showing the average frequency of action potential firing (Hz) at +80 pA current injection in control (n = 26 cells, black bar) and in 1 nM BPA-treated neurons (n = 21 cells, blue bar). (**J**) Bar graph showing the average frequency of action potential firing (Hz) at +100 pA current injection in control (n = 26 cells, black bar) and in 1 nM BPA-treated neurons (n = 21 cells, blue bar) Data are represented as the mean ± s.e.m (N = 8 animals). Student’s *t*-test: **p < 0.01; ***p < 0.001; ns, not significant. (**G**: p = 0.0001, t = 4.197, df = 45; **H**: p = 0.22, Mann-Whitney U = 211.0; **I**: p < 0.0001, Mann-Whitney U = 72.50; **J**: p = 0.001, t = 3.495, df = 41).

To study whether BPA directly affects sensory neurons, we performed current-clamp studies in small-diameter DRG neurons (<30 μm) incubated with 1 nM BPA or vehicle for 24–48 h. BPA had no significant effect on input membrane resistance, input membrane capacitance or action potential amplitude (control, 987 ± 111 MΩ, n = 26 *vs* BPA, 838 ± 81 MΩ, n = 21; control, 12.0 ± 0.6 pF, n = 26 *vs* BPA, 11.1 ± 1.1 pF, n = 21; control, 107.3 ± 3.1 mV, n = 26 *vs* BPA, 97.8 ± 3.8 mV, n = 21, respectively). In contrast, the resting membrane potential of neurons exposed to BPA was depolarized by 5 mV (control, −56.5 ± 1.5 mV, n = 26 *vs* BPA, −51.5 ± 1.8 mV, n = 21; *p < 0.05). We therefore measured the threshold current required to elicit an action potential using a series of 1 s duration current injections, increasing in final amplitude by 10 pA between each successive stimulus. Representative responses of control and BPA-treated neurons to different current inputs are illustrated in Fig. 2A–F. There was a marked reduction of approximately 38% of the current threshold for single action potential generation in BPA-treated neurons (control, 52.7 ± 6.3 pA, n = 26 *vs* BPA, 20.0 ± 3.7 pA, n = 21; ***p < 0.0001; Fig. 2G). In addition, although the frequency of action potential firing at the threshold current was similar in both groups (control, 1.16 ± 0.07 Hz, n = 26 *vs* BPA, 1.55 ± 0.27 Hz, n = 21; Fig. 2H), an injection of +80 pA elicited three times the number of action potentials in BPA compared to vehicle treated neurons (10.8 ± 1.8 Hz, n = 21 for BPA vs 3.2 ± 0.8 Hz, n = 26 for control; ***p < 0.001; Fig. 2I). Similar differences in the frequency of action potential firing were observed for a larger current injection (control, 4.1 ± 1.1 Hz, n = 26 *vs* BPA, 11.0 ± 1.8 Hz, n = 21; **p < 0.001; Fig. 2J).

### Na_v_1.7, Na_v_1.8 and Na_v_1.9 mRNA expression are unaffected by BPA

The Na_v_1.7 channel is expressed in the peripheral nervous system, is sensitive to tetrodotoxin (TTX)^24,25^ and plays a key role in initiating action potentials in nociceptive neurons^26^. In addition to Na_v_1.7, also Na_v_1.8 and Na_v_1.9 are expressed in peripheral neurons and have been linked to pain pathways^27^.

**Figure 3:**
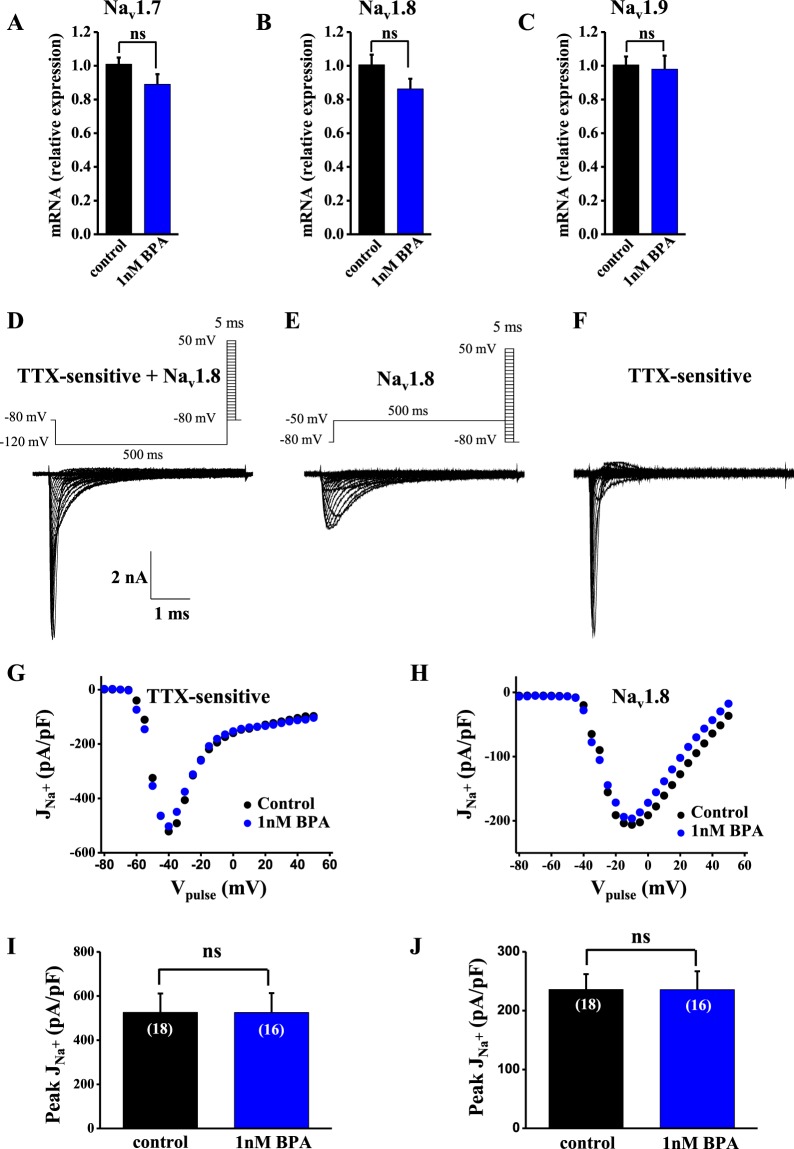
BPA treatment does not affect the expression of sodium channels or the amplitude of TTX-sensitive or Na_v_1.8 currents in DRG neurons. (**A–C**) Bar graphs showing the relative expression of *Scn9a* (Na_v_1.7), *Scn10a* (Na_v_1.8) and *Scn11a* (Na_v_1.9), respectively (in control neurons (black bars) and BPA-treated neurons (blue bars). The mRNA expression of the sodium channels was analyzed by quantitative RT-PCR and was normalized by housekeeping gene *Hprt*. The results were obtained from three different preparations of dissociated DRG neurons from 6 male mice (experiments performed in duplicate). (**D–F**) Representative voltage-clamp current recordings showing TTX-sensitive plus Na_v_1.8 currents, Na_v_1.8 currents and TTX-sensitive currents, respectively. To obtain isolated TTX-sensitive currents, Na_v_1.8 currents were subtracted from TTX-sensitive plus Na_v_1.8 currents. See Methods section for a detailed explanation. Insets in the top of panels D and E show the voltage protocol used. Only the currents obtained with test pulses (−80 to +50 mV, 5 ms) are shown. Calibration bars in (**D**) apply to (**E,F**). (**G,H**) Representative relationship between sodium currents density (**J**, sodium currents normalized to cell size in pF) and the voltage of the test pulse in control (black circles) and 1 nM BPA-treated cells (blue circles) for TTX-sensitive and Na_v_1.8 currents, respectively. (**I,J**) Average peak current density in control cells (black bar; n = 18 cells) and 1 nM BPA-treated cells (blue bar; n = 16 cells) obtained from *J-V* relationships for TTX-sensitive and Na_v_1.8 currents, respectively. Data are represented as the mean ± s.e.m (N = 11 animals). Student’s *t*-test: ns, not significant. (**A**; p = 0.17, t = 1.448, df = 12. **B**; p = 0.13, t = 1.607 df = 12; **C**; p = 0.81, t = 0.244 df = 12).

To examine whether Na_v_1.7, Na_v_1.8 and Na_v_1.9 expression in DRG neurons were affected by BPA, we measured their mRNA levels by quantitative RT-PCR in DRG neurons treated with 1 nM BPA or vehicle for 24–48 h. The results indicate that 24–48 h of 1 nM BPA treatment did not affect voltage-dependent sodium channel gene transcription (Fig. 3A–C).

In addition, we measured the mRNA levels of TRPA1 and TRPV1 in DRG neurons treated with 1 nM BPA, because these TRP channels mediate pain^28^. The expression of these TRP channels also remained unchanged by BPA treatment (Supplemental Fig. 1).

### Amplitude of sodium currents is unaffected by BPA

To study whether BPA functionally affect Na_v_ currents independently of transcriptional effects, we recorded voltage-gated sodium currents using the patch-clamp technique in the whole-cell configuration of small-diameter DRG neurons (<30 μm). Cell size was measured using average capacitance^29^ which was 14.6 ± 1.5 pF for vehicle (n = 18) and 13.8 ± 1.1 pF for BPA-treated group (n = 16). Based on their size, this group of neurons is preferentially nociceptive^26,30,31^. As mentioned above, sodium channels Na_v_1.7, Na_v_1.8 and Na_v_1.9 were expressed in peripheral neurons. To measure the amplitude of TTX-sensitive sodium currents, including a large amount of Na_v_1.7 current, but probably with some Na_v_1.6 current, we combined two electrophysiological protocols^32^. Briefly, an I–V curve family of TTX-sensitive and Na_v_1.8 (TTX-resistant) currents was recorded using a holding potential of −80 mV, which inactivates Na_v_1.9 channels^33^. Because some of the TTX-sensitive current is also inactivated at −80 mV, a long prepulse of 500 ms at a potential of −120 mV was applied to remove the fast inactivation of TTX-sensitive currents, yet maintaining Na_v_1.9 inactivation^33^. This produced an estimation of the total Na^+^ current produced by TTX-sensitive and Na_v_1.8 (Fig. 3D). To reveal the TTX-sensitive current in isolation, a family of currents was recorded using a prepulse of 500 ms to −50 mV, which inactivated the TTX-sensitive current while leaving the Na_v_1.8 current intact (Fig. 3E). Then, we subtracted Na_v_1.8 currents (Fig. 3E) from TTX-sensitive + Na_v_1.8 currents (Fig. 3D) to only visualize TTX-sensitive currents (Fig. 3F). When normalized sodium currents (pA/pF; JNa^+^) were represented *vs* voltage pulse, we observed no significant differences between BPA-treated and vehicle-treated DRG neurons, either for TTX-sensitive or Na_v_1.8 currents (Fig. 3G,H, respectively, representative J-V curves). The threshold activation voltage and the voltage of the peak current were not affected by BPA (Fig. 3G,H), nor was the average peak current densities of either the TTX-sensitive currents or Na_v_1.8 channels (Fig. 3I,J, respectively).

### BPA increased the amplitude of Na_v_ ramp currents

**Figure 4:**
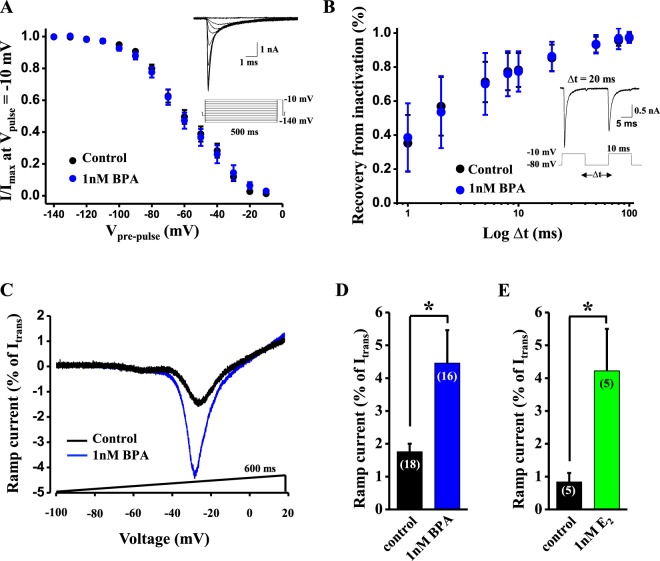
BPA increases the amplitude of Na_v_ ramp currents in DRG neurons. (**A**) Average relationship between normalized sodium currents (I/I_max_) evoked with the test pulse (−10 mV, 5 ms) and the voltage of the prepulse (−140 to −10 mV, 500 ms) for the measurement of the steady-state fast inactivation in control (black circles, n = 18 cells) and 1 nM BPA-treated neurons (blue circles, n = 16 cells). Inset shows the entire voltage protocol (bottom) and the current recorded with the test pulse (−10 mV, 5 ms). (**B**) Average relationship between the recovery from inactivation of sodium currents induced by two identical voltage steps (−80 to −10 mV, 10 ms; measured as a percentage) and the time between these two pulses (∆t, in ms) in control (black circles, n = 18 cells) and 1 nM BPA-treated neurons (blue circles, n = 16 cells). Inset shows the voltage protocol (bottom) and the currents recorded with the two pulses when ∆t = 20 ms. (**C**) Representative recording showing the Na_v_ ramp current in response to a voltage ramp of 600 ms duration from −100 to +20 mV in a control (black trace) and a 1 nM BPA-treated neuron (blue trace). (**D**) Bar graph showing the average Na_v_ ramp currents in control (n = 18 cells, black bar) and 1 nM BPA-treated neurons (n = 16 cells, blue bar) for 24–48 h. (**E**) Bar graph showing the average Na_v_ ramp currents in control (n = 5 cells, black bar) and 1 nM 17-β estradiol (E_2_) (n = 5 cells, green bar) for 24–48 h. Ramp currents in (**C–E**) are expressed as a percentage of the peak of the transient Na_v_ current. Data are represented as the mean ± s.e.m (N = 5 animals, same animals employed in Fig. 3). (**A,B**) Student’s *t*-test: not significant for all points tested. Student’s *t*-test: *p < 0.05. (**D**; p = 0.01, t = 2.610, df = 32. **E**; p = 0.04, t = 2.57, df = 6).

The biophysical properties of Na_v_1.7 make it well-suited for low frequency firing of nociceptive C-fibers because it produces rapidly activating and inactivating currents, yet displays a slow recovery from inactivation. It is of note that Na_v_1.7 is characterized by a slow closed-state inactivation, allowing the channel to produce a ramp current in response to small, slow depolarizations, such as the receptor potentials generated by the by the pain mediators prostaglandins, histamine, ATP and serotonin. The ramp current evoked with the receptor potential depolarizes the cell and increases the probability that the neuron will reach the threshold and fire action potentials^26,34^. Hence, we investigated whether 1 nM BPA directly affected the biophysical properties of Na_v_ currents. Neither the fast inactivation nor the recovery from inactivation were affected by BPA (see Fig. 4A,B). However, 1 nM BPA increased the Na_v_ ramp current in response to a ramp of 600 ms duration from −100 to +20 mV (Fig. 4C). The average ramp current measured at the peak increased from the control level of 1.76 ± 0.25% of I_trans_, n = 18 to 4.46 ± 1.03% of I_trans_, n = 16 for BPA; *p < 0.05; Fig. 4D). This effect was similar when the natural hormone 17β-estradiol was used at 1 nM instead of BPA (Fig. 4E).

### BPA-increased Na_v_ ramp currents involve estrogen receptor ERα

**Figure 5:**
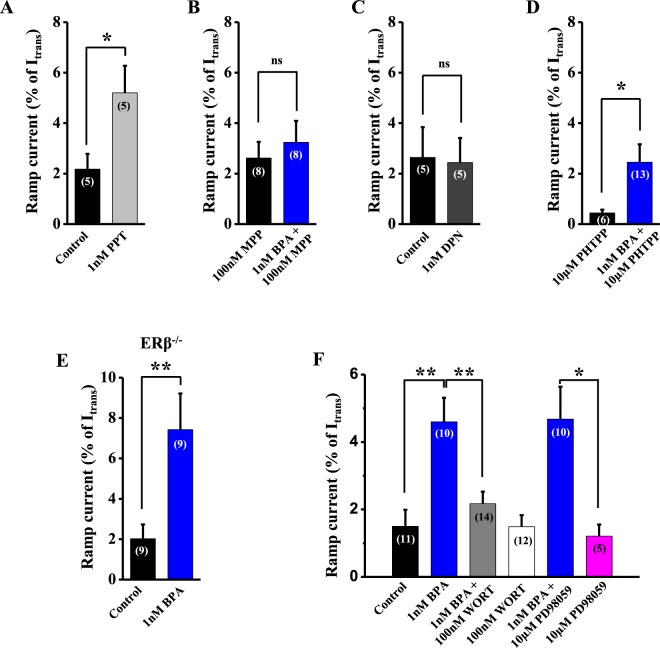
BPA enhances ramp currents through ERα/PI3K signaling pathway. (**A**) Bar graph showing the average Na_v_ ramp currents in control DRG neurons (n = 5 cells, black bar) and in the presence of the specific ERα agonist propyl pyrazole triol (PPT, 1 nM) (n = 5 cells, light gray bar). (**B**) Bar graph showing the average Na_v_ ramp currents in the presence of the specific ERα antagonist methyl-piperidino-pyrazole (MPP, 100 nM) (n = 8 cells, black bar) and in the presence of MPP and 1 nM BPA (n = 8 cells, blue bar) in DRG neurons. (**C**) Bar graph showing the average Na_v_ ramp currents in control (n = 5 cells, black bar) and in the presence of the specific ERβ agonist diarylpropionitrile (DPN, 1 nM) (n = 5 cells, dark-gray bar) in DRG neurons. (**D**) Bar graph showing the average Na_v_ ramp currents in the presence of the specific ERβ antagonist phenyl trifluoromethyl pyrazolo pyrimidin (PHTPP, 10 μM) (n = 6 cells, black bar) and in the presence of PHTPP and 1 nM BPA (n = 13 cells, blue bar) in DRG neurons. (**E**) Bar graph showing the average Na_v_ ramp currents in control (n = 9 cells, black bar) and in the presence of 1 nM BPA (n = 9 cells, blue bar) in DRG neurons from ERβ^−/−^ mice. (**F**) Bar graph showing the average Na_v_ ramp currents in control DRG neurons (n = 11 cells, black bar), in the presence of 1 nM BPA (n = 10 cells, blue bar), in the presence of 1 nM BPA and 100 nM wortmannin (n = 14 cells, gray bar), in the presence of 100 nM wortmannin alone (n = 12 cells, white bar), in the presence of 1 nM BPA and 10 µM PD98059 (n = 10 cells, blue bar) and in the presence of 10 µM PD98059 alone (n = 5 cells, pink bar) in DRG neurons. Ramp currents in all panels are expressed as a percentage of the peak of the transient Na_v_ current. Data are represented as the mean ± s.e.m (N = 11 animals). Panels (A-E): Student’s *t*-test: *p < 0.05; **p < 0.01; ns, not significant. (**A**; p = 0.02, t = 2.747, df = 8. **B**; p = 0.54, t = 0.62 df = 14; **C**; p = 0.88, t = 0.14 df = 8; **D**; p = 0.03, t = 2.27 df = 16; **E**; p = 0.005, t = 3.281 df = 15). Panel (F): one-way ANOVA followed by Holm-Sidak; **p < 0.01, *p < 0.05. (control vs 1 nM BPA: p = 0.003, t = 3.72; 1 nM BPA vs 1 nM BPA + 100 nM WORT: p = 0.02, t = 2.95; 1 nM BPA + 10 µM PD98059 vs 10 µM PD98059; p = 0.02, t = 2.92; control vs 10 µM PD98059: p = 0.50, t = 0.67; control vs 1 nM BPA + 10 µM PD98059; p = 0.02, t = 2.66).

DRGs express estrogen receptors ERα and ERβ^35^. We sought to investigate whether they had a role in the action of BPA on Na_v_ ramp currents. When DRG neurons were incubated with a specific agonist of ERα, propyl pyrazole triol (PPT, 1 nM), the ramp current was statistically increased (control, 2.18 ± 0.6% of I_trans_, n = 5 *vs* PPT, 5.2 ± 1.07% of I_trans_, n = 5; *p < 0.05; Fig. 5A). Furthermore, BPA had no effect of the ramp current in the presence of a specific antagonist of ERα, methyl-piperidino-pyrazole (MPP, 100 nM), (MPP, 2.62 ± 0.64% of I_trans_, n = 8 *vs* MPP + BPA, 3.24 ± 0.85% of I_trans_, n = 8; Fig. 5B). The incubation of DRG neurons with MPP alone had no effect on ramp currents (compare control in Fig. 5A with MPP alone in Fig. 5B). These experiments strongly suggest that ERα rather than ERβ is responsible for the effect of BPA on Na_v_ ramp currents. The lack of contribution of ERβ was further supported by the lack of effect of a specific agonist of ERβ, diarylpropionitrile (DPN, 1 nM) on the ramp currents(control, 2.64 ± 1.2% of I_trans_, n = 5 *vs* DPN, 2.44 ± 0.97% of I_trans_, n = 5; Fig. 5C). Furthermore, 1 nM BPA in the presence of a specific antagonist of ERβ, phenyltrifluoromethyl pyrazolopyrimidine (PHTPP, 10 μM), increased ramp currents (PHTPP, 0.44 ± 0.13% of I_trans_, n = 6 *vs* PHTPP + BPA, 2.45 ± 0.71% of I_trans_, n = 13, *p < 0.05; Fig. 5D). As the Na_v_ ramp current values in the presence of the PHTPP were considerably reduced in comparison to the ramp current values in the rest of the experiments, we wanted to further confirm the lack of participation of ERβ, and the contribution of ERα, in the effect of BPA on Na_v_ ramp currents. To do this, we used DRG neurons from ERβ^−/−^ mice. Incubation of DRG neurons from ERβ^−/−^ male mice with 1 nM BPA increased the ramp current to a similar extent as in wild-type animals (control, 2.02 ± 0.71% of I_trans_, n = 9 *vs* BPA, 7.43 ± 1.79% of I_trans_, n = 9, *p < 0.01; Fig. 5E). Moreover, the ramp currents of DRG neurons from ERβ^−/−^ male mice incubated with the vehicle (control in Fig. 5E) had similar ramp currents to vehicle-treated DRG neurons from wild-type animals (controls in Figs 4D, 5A,C).

### Potentiation of Na_v_ ramp currents by BPA involves phosphoinositide 3-kinase

ERα acts outside the nucleus through activation of PI3K and extracellular signal–regulated kinases (ERK1/2) signaling pathways, among others^36–38^. In pancreatic β-cells, ERα-mediated actions involved these two kinases^15,39^. To investigate whether PI3K plays a role in the potentiation of Na_v_ ramp currents by BPA, we measured the ramp currents in DRG neurons incubated with 1 nM BPA in the presence or absence of the broad-spectrum PI3K inhibitor wortmannin (100 nM). We observed that the BPA-induced potentiation of the ramp currents was strongly reduced by wortmannin (control, 1.50 ± 0.49% of I_trans_, n = 11 *vs* BPA, 4.16 ± 0.71% of I_trans_, n = 10; **p < 0.01 and BPA *vs* wortmannin + BPA, 2.17 ± 0.36% of I_trans_, n = 14; **p < 0.01; Fig. 5F), while wortmannin itself had no effect on the ramp currents (1.49 ± 0.34% of I_trans_, n = 12, Fig. 5F). Furthermore, the effects of BPA on resting membrane potential and firing frequency (see above) were abolished in the presence of wortmannin (Supplemental Fig. 2). This result indicates that the potentiation of Na_v_ ramp currents by BPA involves PI3K. ERK1/2 has also been observed to induce phosphorylation of Na_v_ and modulation of its gating properties, thereby contributing to the regulation of DRG neuron excitability^40^. We therefore assessed the involvement of this cellular pathway in the action of BPA on Na_v_ ramp currents. However, incubation of DRG neurons with the specific ERK1/2 inhibitor 2-(2-Amino-3-methoxyphenyl)-4H-1-benzopyran-4-one (PD98059, 10 μM) had no effect on the BPA-induced potentiation of the ramp currents (control, 2.02 ± 0.42% of I_trans_, n = 9 *vs* PD98059 + BPA, 4.68 ± 0.96% of I_trans_, n = 10; *p < 0.05; PD98059, 1.21 ± 0.33% of I_trans_, n = 5 *vs* PD98059 + BPA, 4.68 ± 0.96% of I_trans_, n = 10; *p < 0.05, Fig. 5F). It should be noted that PD98059 itself had no effect on the ramp currents (PD98059, 1.21 ± 0.33% of I_trans_, n = 5 *vs* control, 2.02 ± 0.42% of I_trans_, n = 9; ns, Fig. 5F).

## Discussion

Exposure to bisphenol A, an endocrine-disrupting chemical, has been linked to several diseases, including disruption of the nervous system^3^. Evidence points to a role of BPA at environmentally relevant doses of 40 μg/kg/day in increasing the perception of noxious stimuli^4^ and under high exposures such as 5 mg/kg/day^41^ and 500 μg/kg/day^5^. The findings described here indicate that subcutaneous injection of BPA, at the tolerable daily intake recommended by the US EPA (50 µg/kg/day), increased the sensitivity to thermal pain in mice treated for eight days using the hot-plate test at a temperature of 50 °C. This exacerbated pain behavior may involve a direct action of BPA on sensory neurons or an effect on the central nervous system, or both. Our results using DRG neurons exposed *in vitro* to 1 nM BPA indicate that primary sensory neurons are indeed directly affected by BPA exposure, although we cannot rule out an effect on supraspinal neuronal circuits involved in pain processing, as reported elsewhere^4^. The fact that in the vehicle-treated group (control) a decrease in the latency to pain response was observed, suggests a habituation to the test, as observed previously^23^. Furthermore, in the days post-treatment that can be observed in Fig. 1 (days 1, 4, 7 and 10 post) no vehicle or BPA was injected to the animals, allowing for a full washout of the compound and confirming the interpretation that the augmented response to pain was dependent on BPA presence. It should be noted that the hot-plate test in the present study did not assess the pain threshold nor use other thermal pain tests, such as the tail-flick response or other assessments of nociceptive mechanical stimuli. Therefore, the fact that the *in vivo* effect of BPA was only assessed with the hot-plate test at a fixed temperature must be considered as a limitation of our study. More extensive future studies of such actions of BPA would be worthwhile to assess pain responses other than thermal pain.

A central observation in the present study is that a concentration of BPA typically found in human blood (1 nM)^42,43^ altered the slow closed-state inactivation voltage-gated Na_v_ channels in cultured primary neurons. The consequence of this effect is to enhance ramp currents in response to small depolarizations and thereby action potential generation, a fundamental role of voltage-gated sodium channels in excitable cells. Genetic, structural and functional studies have shown that the Na_v_1.7 subunit is a major contributor to pain perception^26^. The fact that 1 nM BPA increases the Na_v_ ramp currents in DRG neurons, including Na_v_1.7, implies that the amplification of weak stimuli to reach the threshold for firing action potentials is increased in the neurons bearing Na_v_ channels. As a consequence, the excitability of these neurons is augmented, and then the transmission of the pain signal will be facilitated. This would mechanistically explain our results in *in vivo* experiments in which the thermal pain behavior was increased by BPA injection.

Regarding the rationale of BPA dosing levels, we have selected 50 μg/kg/day for *in vivo* experiment and 1 nM for *in vitro* experiments, as these doses are within the range of human exposure and employed in many studies^13,42,44,45^. We have recently published that BPA affects the electrical activity of the excitable pancreatic β–cell in a non-monotonic dose-response relationship, with 1 nM BPA being the most effective concentration^15^. In addition, in a previous paper of our group, we reported an effect of 1 nM BPA on ion channel gating in pancreatic β–cells from human islets^14^. For these reasons, we feel that the selected doses in the present study are appropriate for a first description of the molecular mechanisms of BPA action in pain signaling.

Although the current work is based upon murine studies, it should be noted that the pharmacokinetics of BPA is similar between mice and humans, with comparable serum-conjugated BPA levels over the 24 h period after BPA administration^46^. It could be argued that oral administration is a more relevant route of exposure to compare with human exposure, however previous work has shown that subcutaneous exposure represents a better means of ensuring a ratio of serum conjugated *vs* unconjugated BPA within the range observed in human studies^6^. Nevertheless, the correlation between BPA dose administered and average serum concentration achieved is not exempt from controversy and remains under study in our laboratory.

As outlined above, small DRG neurons express Na_v_1.8 and Na_v_1.9 in addition to Na_v_1.7. Although Na_v_1.8 produces most of the current underlying the depolarizing phase of action potential^47^, a contribution of Na_v_1.8 to ramp currents has also been reported^48^. However, this contribution of Na_v_1.8 occurs preferentially in human DRG neurons in comparison with rodent species^48^. Na_v_1.6 and Na_v_1.3 channels can also produce a response to slow ramplike stimuli^49–51^. However, Na_v_1.3 channels present a slow inward current separated by two peaks. The first one corresponds to the window current predicted by the overlap of the voltage-dependent activation with the fast inactivation and thus it occurs around −40 mV. The second peak occurs at more depolarized potentials (−20 mV) and it appears to be relatively insensitive to closed-state inactivation^52^. In our present study, only one peak of the ramp current is observed and occurs at −30 mV, ruling out a role for Na_v_1.3. In addition, Na_v_1.3 channels are expressed only in DRG neurons after nerve injury^47^. Na_v_1.6 is highly expressed in large DRG neurons while small DRG neurons express low mRNA levels of this sodium channel^53^. Moreover, the ramp currents generated by Na_v_1.6 channels are smaller than Na_v_1.7 ramp currents^51^. For these reasons, we believe that the effect of BPA on ramp currents of mice DRG small neurons in our study occurs mainly through Na_v_1.7 channels, although we cannot rule out a small contribution of other sodium channels to ramp currents.

**Figure 6:**
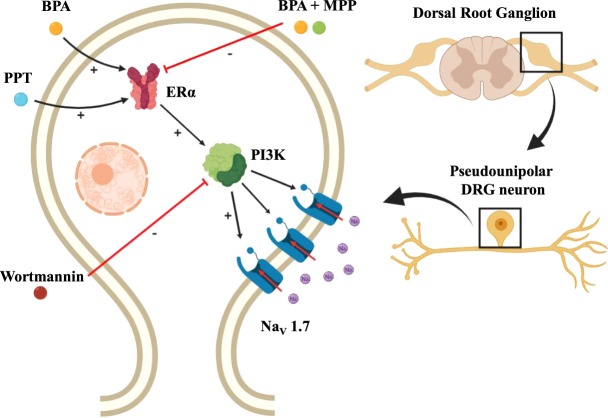
Cellular model of the potentiation of Na_v_1.7 ramp currents in DRG neurons by 1 nM BPA. Binding of BPA to ERα activates PI3K, which in turn modulates Na_v_1.7 channels to increase ramp currents. Inhibition of ERα by MPP or PI3K by wortmannin abolished the effect of BPA. However, ERβ is not implicated in potentiation of Na_v_1.7 ramp current by BPA in DRG neurons, as DPN had no effect and BPA in the presence of PHTPP had effect.

Several reports have shown that estradiol upregulates Na_v_1.7 and other voltage-gated sodium channels in sensory neurons of DRG^54^, human embryonic stem cell–derived sensory neurons^55^ and trigeminal ganglia^12^. This implies a contribution of estradiol to hyperalgesia through a genomic action. In contrast, environmentally relevant doses of BPA did not alter the expression of voltage-gated Na_v_1.7, Na_v_1.8 or Na_v_1.9 sodium channels in the present study. Instead, BPA alters the ramp currents of Na_v_, most likely acting outside of the cell nucleus through activation of PI3K. Several kinases have been reported to be implicated in the regulation of the biophysical properties of Na_v_1.7, as PKA and PKC^56^, ERK1/2 MAP kinase^40^ and tyrosine kinase^57^. Hence, it is tempting to speculate that PI3K may phosphorylate Na_v_ channels altering their gating properties. When BPA acts in its classical manner, that is as a transcription factor via estrogen response elements, it behaves as a weak estrogen, since its binding activity to ERα and to ERβ is over 1000–10,000-fold lower than that of estradiol^58–60^. Indeed, BPA has less efficiency in the activation of ER and recruits lower amounts of transcriptional coregulators in comparison to estradiol, probably due to weaker binding interactions of BPA with ERα^61^. However, an increasing number of studies during the last twenty years have demonstrated that BPA can elicit cellular and physiological effects with the same potency as estradiol, within the nanomolar range to which humans are exposed^44,45,62,63^. These low-dose effects can be explained by the fact that BPA elicits rapid responses via nonclassical estrogen-triggered pathways^45,64–68^. Relevant to our findings, stimulation with estrogens increases ERα-mediated PI3K activity independently of gene transcription^36^ and estradiol can regulate the epithelial sodium channel via a rapid nongenomic mechanism involving the activation of the PI3K signaling pathway^69^. Although it has been reported that E_2_ can also modulate the activity of several ion channels through signaling pathways involving ERK1/2^9,10^, our results with the specific blocker of ERK1/2 (PD98059) indicate that this enzyme is not involved in the action of BPA on Na_v_ ramp currents in DRG neurons. The acute application of 1 nM BPA (7.7 ± 0.8 min) to eight DRG neurons, had no effect on sodium channel ramp currents, indicating that BPA was not exerting a rapid action on sodium channel ramp currents (results not shown). It is possible that BPA is altering the expression of genes other than sodium channels, for example the PI3K gene, through nonclassical estrogen-triggered pathways, and further experiments are required to address this hypothesis. In summary, therefore, the effect of BPA on Na_v_ ramp currents in DRG neurons must be mediated by nonclassical activation of ERα and subsequent activation of PI3K, similar to some of the reported effects of estradiol (Fig. 6).

The fact that our results with BPA are reproduced with 17β-estradiol at the same dose, 1 nM, indicates, a nonclassical action in the enhancement of Na_v_ ramp currents. This estrogenic action may contribute to a greater prevalence in women of a number of pain conditions, including temporomandibular disorder, irritable bowel syndrome, fibromyalgia, and migraine^70,71^, and also help explain why women exhibit also lower pain thresholds^72^.

The voltage-gated sodium channel Na_v_1.7 is expressed in the peripheral nervous system and has a principal role in the pathophysiology of pain^26,73^. Na_v_1.7 is present not only in nociceptors within the dorsal root ganglia but also in sympathetic ganglion neurons^25^, myenteric neurons^74^, olfactory neurons^75^ and pancreatic endocrine cells^76,77^. Hence, a gain of function of this channel induced by BPA could affect not only pain signaling but also autonomous sympathetic functions, such as the control of blood vessel caliber and gastrointestinal function. Indeed, a case report indicates that occupational exposure to BPA as a consequence of facsimile paper production was responsible for inducing the condition of erythema, due to dilated blood vessels and dermatitis^78^. BPA at a no-observed-adverse-effect level (NOAEL: 5 mg/kg/d), has also been reported to influence intestinal barrier function and gut nociception^41^. Furthermore, it has been proposed that mutations of Na_v_1.7 in human pancreatic β-cells may increase susceptibility to development of diabetes via β-cell injury^79^.

Gain-of-function mutations in *SCN9A*, which encodes the voltage-gated Na_v_1.7 channel, produce inherited erythromelalgia^80^ and paroxysmal extreme pain disorder^81^. Erythromelalgia is characterized by the combination of recurrent burning pain, warmth and redness of the extremities, and the mutations in *SCN9A* shift the voltage-dependence of Na_v_1.7 activation in a hyperpolarized direction, increase ramp currents and slow deactivation. Interestingly, these alterations in the biophysical properties of Na_V_1.7 produce hyperexcitability of DRG neurons and hypoexcitability of sympathetic neurons^82^. It is tempting to speculate that the effects of BPA on pain shown in our present results and on erythema^78^ could be induced by a BPA action on Na_v_1.7 ramp currents in both neuronal types. As a result of the biophysical characteristics of Na_v_1.7 channels, some of the gain-of-function mutations of this channel depolarize the resting potential by ~5 mV without a concomitant effect on the input resistance of the neuron^83^. Our results with BPA on DRG neurons produced the same result, suggesting again that the effects of BPA can be explained by a gain of function on Na_v_1.7 ramp currents.

The main mode of action of BPA is as an agonist of estrogen receptors, although antiandrogenic actions and antagonism of thyroid hormone receptors have been reported^84,85^. In our present work, the action of BPA was mediated through activation of ERα, as it was mimicked by the specific agonist of ERα PPT. BPA in the presence of the specific antagonist of ERα MPP had no effect, and the action of BPA was present in DRG neurons from ERβ^−/−^ male mice. These results indicate that the effect of BPA on Na_v_ ramp currents was mediated by ERα with no contribution of ERβ and suggest that the effect of the high concentration of PHTPP, a specific antagonist of ERβ, used in the experiment shown in Fig. 5D probably had some unspecific effect. Although the estrogen receptor GPR30 is also expressed in DRG neurons^86^, the effect of 17β-estradiol through GPR30 is known to inhibit pain^9^. Thus, if BPA is able to activate GPR30 this may limit the extent of the observed increase in nociception and merits future work.

In the present study, we have investigated the action of BPA in biological events at molecular and cellular signaling levels, leading to the adverse outcome of altered pain response in the whole animal. According to our results, an increase in Na_v_ ramp currents induced by 1 nM BPA through ERα-PI3K activation leads to a higher spike firing and then to an increased pain sensitivity. Our results in this particular work followed an Adverse Outcome Pathway (AOP) conceptual framework^87^. We have investigated the action of BPA in biological events at molecular and cellular signaling levels, leading to the adverse outcome of altered pain response in the whole animal.

In summary, the present study extends our knowledge on the effects of BPA on adult neurons. We demonstrate that BPA at environmentally relevant doses affects the ability to detect noxious stimuli, which is essential for the survival of organisms. The nociceptive response to a thermal stimulus is increased in mice treated with BPA at the current safety level set by the US Environmental Protection Agency (50 µg/kg/day). Furthermore, incubation of dorsal root ganglia sensory neurons with 1 nM BPA for 24–48 h alters voltage-gated Na_v_ ramp currents and increases excitability in mouse DRG neurons through the ERα-PI3K pathway. These results should be considered when studying the etiology of pain conditions.

## Methods

### Animals and treatment

Adult male C57BL6 mice aged 3–4 months were used. In order to avoid hormonal fluctuation of estrus cycle we decided to use male mice in this study. This allowed us to prevent possible interactions with BPA because of higher estradiol levels in females and to reduce variability. Animals were purchased from Servicio de Experimentación Animal (SEA), Universidad Miguel Hernandez de Elche (Elche, Spain). Animals were housed (5 mice/cage) in type III cage of polycarbonate plastic (Tecniplast), 530 cm^2^. We used new cages to avoid BPA release as much as posible on heat-treated hardwood bedding, under environmental conditions of 22 °C, and a light cycle of 12 h (8:00 am to 8:00 pm). Mice were maintained on 2014 Teklad Global 14% Protein Rodent Maintenance Diet (Harlan Laboratories, Barcelona, Spain), which was free of alfalfa and soybean meal, with *ad libitum* access to food and tap water. The composition of the diet is as follows: crude protein, 14.3%; fat, 4%; carbohydrate, 48%; crude fiber, 4.1%; neutral detergent fiber, 18%; ash, 4.7%; energy density, 2.9 kcal/g (12.1 kJ/g); calories from protein, 20%; calories from fat, 13%; and calories from carbohydrate, 67%. ERβ^−/−^ mice were generated as described elsewhere^88^ and were supplied originally by the laboratory of Jan-Ake Gustafsson. All genetically modified animals and wild types were from the same supplier and the same colony (C57BL6), which is grown in Servicio de Experimentación Animal (SEA), Universidad Miguel Hernandez de Elche. Experimental procedures were reviewed and approved by the institutional committee for animal care and use of the Universidad de Alicante (UA-2017-06-29) and all methods were performed in accordance with the relevant guidelines and regulations. Animals were treated humanely and with regard for alleviation of suffering. For behavioral testing (see below) BPA was dissolved in tocopherol-stripped corn oil and administered subcutaneously for 8 days in a volume of 100 µL. The daily dose used was 50 µg/kg/day. Control animals received a daily dose of vehicle. For patch clamp experiments, dorsal root ganglia (DRG) were extracted from untreated mice. In these experiments, BPA or vehicle (DMSO 0.000004% v/v) were applied only *in vitro* after DRG extraction, during DRG neurons culture (see below).

### Behavioral testing

We used constant-temperature hot-plate measurements (World Precision Instruments, FL). This equipment consisted of a metal plate heated to a preset temperature and a Plexiglass observation chamber. The applied temperature was 50 °C, well above the noxious heat threshold. The holding accuracy of the apparatus is ±0.1 °C as indicated by the manufacturer. We measured the reflex latency of the withdrawal reaction, consisting in paw shaking, licking or escape behavior, such as a jump (whichever came first). Mice were habituated to the test apparatus for at least 30 min before testing on each test day and three repeated tests were performed in this period. 20 mice were used for this test, 10 mice treated with BPA and 10 mice treated with vehicle. A cut-off for maximal exposure to the hot plate was set at 45 s^89^. The reflex latency values were in the range of observed elsewhere^89,90^ ≈20 s. An increase in latency was considered analgesia, while a decrease in latency was regarded as hyperalgesia.

### Primary DRG culture

Previously described culture methods for DRG neurons in adult male mice were used^91,92^. Briefly, 36 male C57BL6 mice were sacrificed using carbon dioxide before confirmation of death, rather than cervical dislocation, as the latter may damage cervical DRG. One culture was generated per mouse. Usually, 6–9 wells were obtained from each culture and a maximum of 3 neuronal recordings were obtained from each well. For the experiments with ERβ^−/−^ 3 mice were used. The vertebral column was quickly removed and dissected in ice-cold Hanks’s balanced sodium salt (Sigma Aldrich, Spain). All DRGs from cervical to sacral segments in both sides of the spinal cord were rapidly removed and enzymatically digested at 37 °C for 15–16 min in INCmix solution containing 900 UI/ml collagenase type XI and 3 mg/ml dispase (Gibco, Thermo Fisher Scientific). After washing with fresh, enzyme-free DRG medium, single neurons were obtained by gentle agitation through a Pasteur pipette. The dissociated DRG neurons were plated into 3.5-cm culture dishes and incubated at 37 °C and 5% CO_2_ in the presence of 1 nM BPA or vehicle (control) for 24–48 h. For the experiments with 1 nM 17-β estradiol (E_2_), the same protocol was employed.

### Patch-clamp recordings

A total of 226 small-diameter (<30 μm) DRG neurons were used for voltage and current-clamp recordings within 24–48 h in culture with 1 nM BPA. This includes 18 neurons from ERβ^−/−^ mice and 10 neurons for experiments with 1 nM E_2_. Cells devoid of processes were selected to avoid space-clamp problems. Patch pipettes were pulled from borosilicate capillaries (World Precision Instruments, FL) using a Flaming/Brown micropipette puller P-97 (Molecular Devices, CA) and heat-polished at the tip using an MF-830 microforge (Narishige, Japan). Electrodes had a resistance of 1–3 MΩ when filled with the pipette solution. For voltage-clamp recording, pipette solutions contained (in mM): 140 CsF, 10 NaCl, 4 Mg-ATP, 0,4 Na-GTP, 1 EGTA, 10 HEPES, 5 Glucose, pH 7.30 (adjusted with CsOH). For current-clamp experiments, pipette solutions contained (in mM): 140 KCl, 10 NaCl, 4 Mg-ATP, 0,4 Na-GTP, 10 HEPES, 5 Glucose, pH 7.30 (adjusted with KOH). For voltage-clamp recordings, the extracellular solution contained the following (in mM): 70 NaCl, 70 Choline Chloride, 3 KCl, 1 MgCl_2_ * 6H_2_O, 1 CaCl_2_, 20 TEA-Cl, 5 CsCl_2_, 0.1 CdCl_2_, 10 HEPES, 10 Glucose, pH 7.4 (adjusted with NaOH). For current-clamp experiments, the extracellular solution contained (in mM): 140 NaCl, 3 KCl, 2.4 CaCl_2_, 10 HEPES, 10 Glucose, pH 7.4 (adjusted with NaOH). Propyl pyrazole triol (PPT, 1 nM) was used as a specific agonist of ERα and methyl-piperidino-pyrazole (MPP, 100 nM) as a specific antagonist of ERα. Diarylpropionitrile (DPN, 1 nM) was used as a specific agonist of ERβ and phenyltrifluoromethyl pyrazolopyrimidine (PHTPP, 10 μM) as a specific antagonist of ERβ. Wortmannin (100 nM) was used as a broad-spectrum PI3K inhibitor. 2-(2-Amino-3-methoxyphenyl)-4H-1-benzopyran-4-one (PD98059, 10 μM) was used as a specific ERK1/2 inhibitor. PPT, MPP, PHTPP, Wortmannin, PD98059 and DPN were obtained from Tocris Cookson Ltd (Avonmouth, United Kingdom). 17-β estradiol used in Fig. 4 was purchased from Sigma-Aldrich (Madrid, Spain).

Whole-cell recordings in voltage-clamp mode were performed with an Axopatch 200B amplifier (Molecular Devices, CA). Data were collected via Digidata 1320 A/D converter (Molecular Devices, CA) at 50 kHz and filtered at 5 kHz. Voltage and current errors were minimized with 80–90% series resistance compensation and linear leak currents, and capacitance artifacts were subtracted out using the P/6 method. The amplitude of TTX-sensitive sodium channels was estimated via two activation protocols. An I–V curve family of TTX-sensitive plus Na_v_1.8 currents were evoked by a series of depolarizing steps from −80 to +50 mV (in increments of 5 mV) at a holding potential of −80 mV, which inactivates Na_v_1.9 channel activity. Because some of the TTX-sensitive current is also inactivated at this voltage, a prepulse of 500 ms to −120 mV was applied to remove the fast inactivation of these currents, although the inactivation of Na_v_1.9 persists over this time. This produced an estimation of the total sodium current in the neuron minus the Na_v_1.9 current. To reveal the TTX-sensitive current in isolation, a family of currents was recorded using a prepulse of 500 ms to −50 mV before the activating pulse in order to inactivate the TTX-sensitive current while leaving the Na_v_1.8 current intact. This family of currents could then be subtracted from the total current in the neuron to view only the TTX-sensitive currents^32^. Ramp currents are expressed as a percentage of the ramp current amplitude in relation to the Na_v_ maximum transient peak current evoked with the protocol explained above for each neuron.

### Real-time PCR

Three different preparations of dissociated DRG neurons from 6 male mice were treated with BPA (1 nM) or vehicle for 24–48 h (measurements performed in duplicate). RNA was extracted using a commercial kit (RNeasy Micro kit, Qiagen, Valencia, CA) according to the manufacturer’s instructions. RNA (0.5 μg) was reverse-transcribed using the High Capacity cDNA Reverse Transcription kit (Applied Biosystems, Foster City, CA). Quantitative PCR (RT-PCR) assays were performed using a CFX96 Real Time System (Bio-Rad, Hercules, CA). Amplification reactions were carried out in medium containing 200 nM of each primer, 1 μl cDNA, and 1x IQ^TM^ SYBR® Green Supermix (Bio-Rad). Hypoxanthine guanine phosphoribosyltransferase (Hprt) was used as the normalization gene. (RT-PCR primers, see Supplementary Fig. 3).

Samples were subjected to the following conditions: 3 min at 95 °C, 40 cycles (5 s at 95 °C, 5 s at 60 °C, and 10 s at 72 °C), and a melting curve of 65–95 °C. Genes were considered as positively amplified when the Ct values were lower than 35 (Ct cut-off value = 35 cycles). The resulting values were analyzed with CFX Manager Version 1.6 (Bio-Rad) and are expressed as the relative expression with respect to control values (2^−ΔΔCT^)^93^.

### Statistical analysis

For statistical analysis, GraphPad Prism (GraphPad software, Inc., CA), Sigmaplot 12.0 (Systat Software, Inc.) or SPSS 22.0 software (Statistical Package for Social Sciences, Chicago, IL, USA) were used. Data are expressed as the mean ± SEM. To assess differences between exposure groups, we used unpaired Student’s test. When data did not pass the parametric test, the Mann-Whitney or Kruskal-Wallis test was used. In Fig. 1 a one-way ANOVA for repeated measures was applied to assess the differences between treatments (vehicle and BPA), as well as any interactions between data of one treatment. Post-hoc pairwise comparisons using Bonferroni’s test were carried out when a 0.05 level of significance was obtained. Values of P < 0.05 were considered to be statistically significant. In Fig. 5F, we used one-way ANOVA followed by the Holm-Sidak test to compare control group *vs* the rest of experimental groups.

## Supplementary information

Supplemental figure 1

Supplemental figure 2

Supplemental figure 3
