# Supplementary material for: Bisphenol A Regulates Sodium Ramp Currents in Mouse Dorsal Root Ganglion Neurons and Increases Nociception
Source: Sci Rep. 2019 Jul 16;9:10306. doi: 10.1038/s41598-019-46769-6 (PMC6635372; doi:10.1038/s41598-019-46769-6)
Supplement: Supplementary file 1 — Supplemental figure 1 [file 41598_2019_46769_MOESM1_ESM.pdf]

# BISPHENOL A REGULATES SODIUM RAMP CURRENTS IN MOUSE DORSAL ROOT GANGLION NEURONS AND INCREASES NOCICEPTION

**Sergi Soriano[1,2*], Minerva Gil-Rivera[1], Laura Marroquí[2], Paloma Alonso-Magdalena[2], Esther Fuentes[2], Jan-Ake Gustafsson[3,4], Angel Nadal[2], Juan Martinez-Pinna[1, 2*]**

[1]Departamento de Fisiología, Genética y Microbiología, Universidad de Alicante, Alicante, Spain.

[2]Institute of Research, Development and Innovation in Biotechnology of Elche (IDiBE), Institute of Molecular and Cellular Biology (IBMC) and CIBERDEM, Miguel Hernández University of Elche, Elche, Alicante, Spain.

[3]Department of Biology and Biochemistry, Center for Nuclear Receptors and Cell Signaling, University of Houston, Houston, Texas, USA.

[4]Department of Biosciences and Nutrition, Karolinska Institut, Huddinge, Sweden.

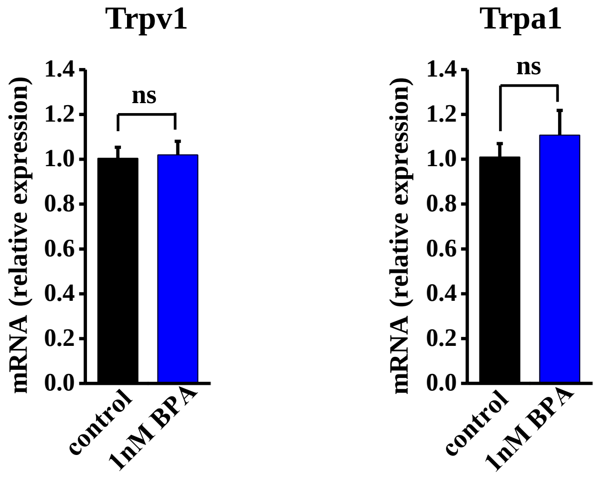


**Supplemental figure 1.** BPA treatment does not affect the expression of TRPV1 and TRPA1 channels.(A, B) Bar graphs showing the relative expression of *Trpv1* and *Trpa1, respectively,* in control neurons (black bars) and in the presence of 1 nM BPA (blue bars) over 24-48 h in DRG neuron. The mRNA expression of the TRP channels was analyzed by quantitative RT-PCR and was normalized by housekeeping gene *Hprt*. (RT-PCR primers, see Supplementary Table 1).The results were obtained from three different preparations of dissociated DRG neurons from 6 male mice (experiments performed in duplicate). (Figure S1 *Trpv1*; $p = 0.78$, $t=0.28$ $df = 12$; Figure S1 *Trpa1*; $p = 0.47$, $t=0.74$ $df = 12$).
