# Supplementary material for: Bisphenol A Regulates Sodium Ramp Currents in Mouse Dorsal Root Ganglion Neurons and Increases Nociception
Source: Sci Rep. 2019 Jul 16;9:10306. doi: 10.1038/s41598-019-46769-6 (PMC6635372; doi:10.1038/s41598-019-46769-6)
Supplement: Supplementary file 2 — Supplemental figure 2 [file 41598_2019_46769_MOESM2_ESM.pdf]

BISPHENOL A REGULATES SODIUM RAMP CURRENTS IN MOUSE DORSAL ROOT GANGLION NEURONS AND INCREASES NOCICEPTION

Sergi Soriano[1,2*], Minerva Gil-Rivera[1], Laura Marroquí[2], Paloma Alonso-Magdalena[2], Esther Fuentes[2], Jan-Ake Gustafsson[3,4], Angel Nadal[2], Juan Martinez-Pinna[1, 2*]

[1]Departamento de Fisiología, Genética y Microbiología, Universidad de Alicante, Alicante, Spain.

[2]Institute of Research, Development and Innovation in Biotechnology of Elche (IDiBE), Institute of Molecular and Cellular Biology (IBMC) and CIBERDEM, Miguel Hernández University of Elche, Elche, Alicante, Spain.

[3]Department of Biology and Biochemistry, Center for Nuclear Receptors and Cell Signaling, University of Houston, Houston, Texas, USA.

[4]Department of Biosciences and Nutrition, Karolinska Institut, Huddinge, Sweden.

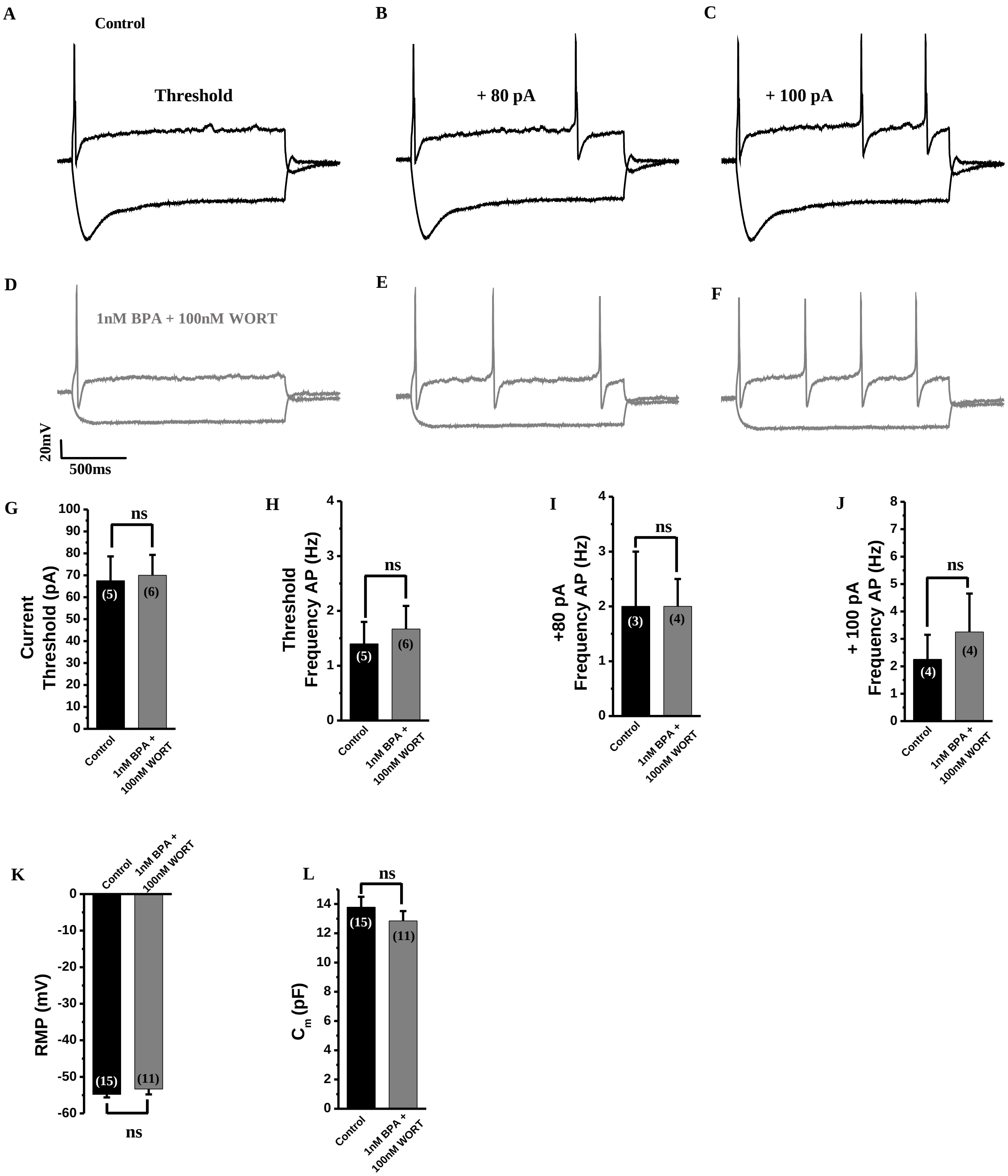

A
Control
Threshold
B
+ 80 pA
C
+ 100 pA
D
1nM BPA + 100nM WORT
E
F
20mV
500ms
G
Current Threshold (pA)
ns
(5)
(6)
Control
1nM BPA + 100nM WORT
H
Threshold Frequency AP (Hz)
ns
(5)
(6)
I
+80 pA Frequency AP (Hz)
ns
(3)
(4)
J
+ 100 pA Frequency AP (Hz)
ns
(4)
(4)
K
RMP (mV)
(15)
(11)
ns
L
C$_m$ (pF)
ns
(15)
(11)

Supplemental figure 2. Wortmannin treatment prevents the effect of BPA in DRG neurons. Representative voltage traces from small-diameter DRG neurons (< 30 µm) in current-clamp experiments in control conditions (A, B, C; black traces) and in the presence of 1 nM BPA + 100 nM Wortmannin for 24-48 h (D, E, F; gray traces) in response to different levels of current injection (threshold, +80 and +100 pA). (G) Bar graph showing the average current threshold (pA) for action potential firing in control (n=5 cells, black bar) and in 1 nM BPA + 100 nM Wortmannin-treated neurons (n=6 cells, gray bar). (H) Bar graph showing the average frequency of action potential firing (Hz) at current threshold injection in control (n= 5 cells, black bar) and in 1 nM BPA + 100 nM Wortmannin-treated neurons (n=6 cells, gray bar). (I) Bar graph showing the average frequency of action potential firing (Hz) at +80 pA current injection in control (n=3 cells, black bar) and in 1 nM BPA + 100 nM Wortmannin-treated neurons (n=4 cells, gray bar). (J) Bar graph showing the average frequency of action potential firing (Hz) at +100 pA current injection in control (n=4 cells, black bar) and in 1 nM BPA + 100 nM Wortmannin-treated neurons (n=4 cells, gray bar). (K) Bar graph showing the Resting Membrane Potential (RMP) in control (n=15, black bar) and in presence of 1 nM BPA + 100 nM Wortmannin-treated neurons (n=11, gray bar). (L) Bar graph showing the average cell membrane capacitance in control (n= 15, black bar) and in presence of 1 nM BPA + 100 nM Wortmannin-treated neurons (n=11, gray bar). Data are represented as the mean ± s.e.m (N=2 animals). Student's t-test: ns, not significant.
