# Supplementary material for: Bisphenol A Regulates Sodium Ramp Currents in Mouse Dorsal Root Ganglion Neurons and Increases Nociception
Source: Sci Rep. 2019 Jul 16;9:10306. doi: 10.1038/s41598-019-46769-6 (PMC6635372; doi:10.1038/s41598-019-46769-6)
Supplement: Supplementary file 3 — Supplemental figure 3 [file 41598_2019_46769_MOESM3_ESM.pdf]

BISPHENOL A REGULATES SODIUM RAMP CURRENTS IN MOUSE DORSAL ROOT GANGLION NEURONS AND INCREASES NOCICEPTION

Sergi Soriano[1,2*], Minerva Gil-Rivera[1], Laura Marroquí[2], Paloma Alonso-Magdalena[2], Esther Fuentes[2], Jan-Ake Gustafsson[3,4], Angel Nadal[2], Juan Martinez-Pinna[1, 2*]

[1]Departamento de Fisiología, Genética y Microbiología, Universidad de Alicante, Alicante, Spain.

[2]Institute of Research, Development and Innovation in Biotechnology of Elche (IDiBE), Institute of Molecular and Cellular Biology (IBMC) and CIBERDEM, Miguel Hernández University of Elche, Elche, Alicante, Spain.

[3]Department of Biology and Biochemistry, Center for Nuclear Receptors and Cell Signaling, University of Houston, Houston, Texas, USA.

[4]Department of Biosciences and Nutrition, Karolinska Institut, Huddinge, Sweden.

Supplementary Figure 3. Primers used in the present study.

Gene	Sequence ID	Sequence (5'-3')
Scn11a **(Nav1.9)**	KM373701.1	FW:ATCCCAAGGCCCCTGAACAAA<br>RV:GTGTGGGCGGGAAGACGTTG
Scn10a **(Nav1.8)**	KM373698.1	FW:ACCGACAATCAGAGCGAGGAG<br>RV:ACAGACTAGAAATGGACAGAATC
Scn9a **(Nav1.7)**	KM373696.1	FW:CCAACCAGTCACCACTCAGC<br>RV:GGGGTCTATGGGGTACAAA
Hprt	NM_013556.2	FW:GGTTAAGCAGTACAGCCCCA<br>RV:TCCAACACTTCGAGAGGTCC
Trpv1	NM_001001445.2	FW:CCGTGTCACTGGAGAGATCC<br>RV:GCCTCTGCAGGAAATACTGG
Trpa1	NM_177781.5	FW:AAACATTGACACATGCTTGGA<br>RV:TTTTCCAAGAGGGAAGTGAGG
